# The WHO global reference list of 100 core health indicators: the example of Sierra Leone

**DOI:** 10.11604/pamj.2017.27.246.11647

**Published:** 2017-08-03

**Authors:** Reinhard Kaiser, Natalie Johnson, Mohamed Falilu Jalloh, Foday Dafae, John Terrell Redd, Sara Hersey, Amara Jambai

**Affiliations:** 1 Centers for Disease Control and Prevention (CDC) Country Office, Freetown, Sierra Leone; 2 Division of Global Health Protection, Center for Global Health, Centers for Disease Control and Prevention, Atlanta, USA; 3 Ministry of Health and Sanitation, Freetown, Sierra Leone

**Keywords:** Sierra Leone, health indicators, health reporting, health surveillance

## Abstract

The global reference list of 100 core health indicators is a standard set of indicators published by the World Health Organization in 2015. We reviewed core health indicators in the public domain and in-country for Sierra Leone, the African continent and globally. Review objectives included assessing available sources, accessibility and feasibility of obtaining data and informing efforts to monitor program progress. Our search strategy was guided by feasibility considerations targeting mainly national household surveys in Sierra Leone and topic-specific and health statistics reports published annually by WHO. We also included national, regional and worldwide health indicator estimates published with open access in the literature and compared them with cumulative annual indicators from the weekly national epidemiological bulletin distributed by the Sierra Leone Ministry of Health and Sanitation. We obtained 70 indicators for Sierra Leone from Internet sources and 2 (maternal mortality and malaria incidence) from the national bulletin. Of the 70 indicators, 14 (20%) were modified versions of WHO indicators and provided uncertainty intervals. Maternal mortality showed considerable differences between 2 international sources for 2015 and the most recent national bulletin. We were able to obtain the majority of core indicators for Sierra Leone. Some indicators were similar but not identical, uncertainty intervals were limited and estimates differed for the same year between sources. Current efforts to improve health and mortality surveillance in Sierra Leone will improve availability and quality of reporting in the future. A centralized core indicator reporting website should be considered.

## Introduction

The global reference list of 100 core health indicators is a standard set of indicators published by the World Health Organization (WHO) in 2015 [1]. The list contains indicators of relevance to country, regional and global reporting across the spectrum of global health priorities relating to the post-2015 health-specific elements of the Sustainable Development Goals (SDG) [1, 2]. The list is divided into 4 domains (health status, risk factors, service coverage and health systems) and 8 subdomains (communicable diseases, reproductive, maternal, newborn, child and adolescent health, non-communicable diseases, injuries and violence and the environment). The main objectives of the reference list are to guide monitoring of health results nationally and globally; to reduce excessive and duplicative reporting requirements; to enhance efficiency of data collection investments; to enhance availability and quality of data and to improve transparency and accountability. The reference list is meant to promote greater alignment with and investment in, one country-led health sector platform for results and accountability that forms the basis for global reporting [3]. In order to provide national data for such a platform, various data sources and data collection methodologies will be necessary to provide the core indicators: civil registration and vital statistics systems; periodic population-based health surveys; facility-generated data that include routine facility information systems and health; facility assessments and surveys; administrative data sources such as financial and human resources information systems; and indicators from other sources, including modeling [1].

Most health indicators in developing countries are currently obtained through national demographic and health surveys (DHS), multiple-indicator cluster surveys, one-time ecologic or household surveys, verbal autopsies, demographic surveillance systems within selected populations [4] and modeling based on trends from regularly repeated surveys. Sierra Leone has made great progress in strengthening its integrated disease surveillance and response (IDSR) system, whose results are regularly published in a weekly bulletin [5]. A broad set of national health monitoring indicators is currently under discussion, including the 100 WHO core indicators. Strengthening epidemiological surveillance and information management, which is an essential component of the national Ebola recovery strategy [6], will contribute to providing data for regular reporting of indicators. The 2016 National Civil Registration Act will establish a new authority in Sierra Leone that will be responsible for registration of citizens and residents and recording vital events including births and deaths [7]. Until these and other improvements in data availability are fully implemented, decision makers will have to rely on international sources for most indicators. We reviewed current availability of the core health indicators in the public domain for Sierra Leone, the WHO African Region (or Sub-Saharan Africa) and the global level for the most recent year and compared them, where available, with indicators published in the most recent Sierra Leone national weekly epidemiological bulletin [5]. The objectives of the review were to assess available sources, accessibility of indicators through the Internet and feasibility of obtaining the data and to inform efforts to monitor program progress.

## Methods

To reduce dependency on the Internet we prioritized compilation reports of health-related data and scientific literature that could be downloaded from the Internet with a locally purchased third-generation (3G) USB modem and reviewed off-line. If indicators were not available in Internet-accessible print versions, we used online databases. For example, we prioritized WHO's World Health Statistics Reports over WHO's online database Global Health Observatory [8]. All reports and scientific articles used for the review were open access. We searched for recent national household surveys in Sierra Leone and topic-specific and health statistics reports published annually by WHO. We also searched for national, regional and worldwide health indicator estimates published with open access in the literature. If provided by the source, we included uncertainty intervals independent of the method used to calculate them and the terminology used (e.g. credible interval, confidence interval). If sources provided values for both sexes and/or breakdowns for male and female, these were presented. If a compilation report provided multiple year ranges instead of a singular year for the indicator we did not attempt to identify a specific year for Sierra Leone from the original data source. Two indicators from the national weekly epidemiological bulletin for epi week 52, 2016 (26 December 2016 to 1 January 2017) were selected to be reported in addition to the most recent Internet source: maternal mortality (annual ratio based on 229,000 live births in 2015 [9]) and malaria (cumulative annual incidence week 1 to 52, 2016 presented in the bulletin). We provided SDG targets [2] and indicated if core and source indicators were not identical but similar and could be considered for adaptation to the core indicator in the future.

## Current status of knowledge

The main country-specific data source used for Sierra Leone was the 2013 DHS [10]. However, most indicators reported for Sierra Leone were available from international compilation reports for 2015. Reports across topic areas were the World Health Statistics Reports 2015 [11] and 2016 [12]. We used topic-or group specific reports on child [13] and maternal mortality [14], non-communicable diseases [15], diabetes [16], malaria [17], road safety [18], sexually transmitted infections [19], suicide [20] and tuberculosis 2015 [21] and 2016 [22]. Information from online databases was used for air pollution [23], cancer [24], International Health Regulations (IHR)-notifiable diseases [25] and immunizations [9, 26]. We included several articles from the Global burden of disease (GBD) Study 2015 [27–29] and an article on trends in diabetes [30]. The national epidemiologic bulletin [5] was obtained from the Ministry of Health and Sanitation in Sierra Leone and was the only document not available on the Internet. Downloading reports and accessing online databases was feasible; however, one of the article supplements [27] had a file size of 295 MB and took about 20 minutes to download. We obtained 70 indicators for Sierra Leone from Internet sources and two (maternal mortality and malaria incidence) from the national weekly epidemiological bulletin (annex). Of those, 42 (60%) were available from 10 international sources [11–13, 15–17, 21, 22, 27–29] and another 17 (24%) were obtained from DHS 2013 [10].

The availability of indicator information by domain was 24 of 27(89%) for health status, 20 of 21(95%) for risk factor, 17 of 27(63%) for health service and 6 of 24(25%) for health systems indicators. Of those reported in the domains, two (8%), six (30%), four (24%) and two (22%), respectively, were modified versions of WHO indicators. Main modifications were either wording differences (9 (64%), e.g. improved vs. safely-managed water, clean vs. modern fuels, married women vs. women of reproductive age who are sexually active); age group differences (4(29%), e.g. adults 15-49 years vs. general population; ≥15 years vs. ≥ 18 years; or both (1(7%)). Of 70 reported indicators for Sierra Leone, 14(20%) included an uncertainty interval; of those, 9 (64%) were health status indicators. A comparison with an estimate for the WHO African Region, Sub-Saharan Africa and/or a global figure was available for 58 (83%) indicators; most indicators without comparison (66%) were from the Sierra Leone DHS 2013 [10]. We identified sources for the majority of indicators recommended by WHO for monitoring health status, risk factors and health services, while only one in four health system indicators could be obtained. We also reported 2 indicators for Sierra Leone's national bulletin (maternal mortality, malaria incidence). Comparisons with African and/or global figures were available for the majority of indicators from Sierra Leone. The review provided the following considerations and lessons learned. First, it was feasible to obtain most core indicators for Sierra Leone through using a locally purchased Internet modem. Other countries may be able to do similar exercises. Second, a limited number of indicators were modified versions of WHO indicators and should be adapted to the core indicator reference list during future data collections.

Third, uncertainty levels were only available for a limited number of indicators, indicating a need to systematically provide those in topic-specific and compilation reports. Forth, different sources reported considerably different estimates for the same indicator and year, reemphasizing the importance of continued efforts to improve health and mortality surveillance in Sierra Leone. As an example, maternal deaths have been added to the IDSR system as a reportable priority event. However, reporting is currently limited to health facilities, likely resulting in considerable underestimation of maternal mortality. The 2015 estimates for maternal mortality in Sierra Leone vary substantially between the WHO (1360 per 100,000 live births, 80% uncertainty interval (UI) 1999-1980) [14] and GBD study (695.7 per 100,000 live births, 95% UI 321.9-1229.1) [29]. The GBD study reported that models were based on more data sources and total site years: globally 599 sources covering 12,052 site years compared to 203 sources covering 2636 total site years in the WHO analysis, for sub-Saharan Africa 124 sources compared to 64 and for Sierra Leone four sources compared to two used by WHO [29].

A more advanced maternal mortality surveillance system would allow to increase accuracy and reliability of estimates and would, combined with availability of cause of death data, allow to inform planning of interventions to reduce maternal mortality in the country, which is among the highest worldwide. Fifth, about one third of the core indicators were unavailable, most of them in the area of health systems. Decision makers should use the results of the review to identify future sources of indicators that were not available or only available in a modified version, including existing surveillance systems and regular national-level surveys. Our review had some limitations. The majority of sources were health reports from the WHO and partner organizations that were accessible in the public domain. We did not attempt to identify other potential sources. Inclusion of indicators with differences in wording or age groups was based on author judgement. Our understanding of how the Ebola outbreak of 2013-2016 affected health indicators in Sierra Leone and the African continent was constrained by a lack of data [31]. Strategizing about how the 30% of indicators that were not available for reporting might be obtained in the future and when and how the 100 indicators will be primarily available from data directly collected from national sources instead of estimates from international sources, was beyond the scope of this article.

## Conclusion

We were able to obtain the majority of core indicators for Sierra Leone. Some indicators were similar but not identical, uncertainty intervals were often not provided and estimates differed for the same year between sources. We think that our review of WHO core indicators in Sierra Leone, which may be the first of its kind in Africa, was a useful exercise that can help countries make their own plans when considering introduction of the core indicators in their health reporting system. Current efforts to improve health and mortality surveillance in Sierra Leone will improve availability and quality of reporting in the future. A centralized, annually updated website for all countries, WHO regions and the global level would facilitate the implementation process and may encourage use of the core indicators.

## Annex

**Annex 1:** Core Health Indicators for Sierra Leone, the WHO African Region and globally, as of January 2017 (**869 kb**)

### What is known about this topic

- A global reference list of 100 core health indicators has been published by the World Health Organization in 2015, however documented experience with the implementation is lacking.

### What this study adds

- To our knowledge, we report the first country review of availability of sources, accessibility of indicators through the Internet, and feasibility of obtaining the data;
- We were able to obtain the majority of core indicators for Sierra Leone: some indicators were similar but not identical, uncertainty intervals were often not provided, and estimates differed for the same year between sources;
- Results of this review can be used to improve health reporting and inform efforts to monitor program progress in Sierra Leone, and help other countries developing their own plans when considering introduction of the core indicators in their reporting system.

## Competing interests

The authors declare no competing interests.

## References

[1] World Health Organization Global Reference List of 100 Core Health Indicators..

[2] The Global Goals The Global Goals for Sustainable Development..

[3] World Health Organization (2011). Monitoring, evaluation and review of national health strategies. A country-led platform for information and accountability..

[4] Larson C, Alec Mercer A (2004). Global health indicators: an overview. Canadian Medical Association Journal..

[5] Ministry of Health and Sanitation, Sierra Leone (2016). Integrated Disease Surveillance and Response. National weekly epidemiological bulletin..

[6] Government of Sierra Leone National Ebola Recovery Strategy for Sierra Leone 2015-2017..

[7] Government of Sierra Leone (2016). National Civil Registration Act 2016, supplement to Sierra Leone Gazette Vol. CXLVII..

[8] World Health Organization Global Health Observatory..

[9] World Health Organization Immunization, vaccines and biologicals. Data, statistics and graphics: information by WHO Member State, WHO Region and on a global level..

[10] Demographic and Health Survey Program Demographic and Health Survey Sierra Leone 2013.

[11] World Health Organization World Health Statistics Report 2015.

[12] World Health Organization World Health Statistics Report 2016.

[13] UN Inter-agency Group for Child Mortality Estimation Levels and trends in child mortality 2015.

[14] World Health Organization Trends in Maternal Mortality: 1990 to 2015.

[15] World Health Organization Global Status Report on Non-communicable Diseases 2014.

[16] World Health Organization Global Report on Diabetes 2015.

[17] World Health Organization World Malaria Report 2015.

[18] World Health Organization Global status report on road safety 2015.

[19] World Health Organization Global incidence and prevalence of selected curable sexually transmitted infections 2008.

[20] World Health Organization Preventing suicide: a global imperative. 2014.

[21] World Health Organization Global TB Report 2015.

[22] World Health Organization Global TB Report 2016.

[23] World Health Organization Global Urban Ambient Air Pollution Database (update 2016.

[24] World Health Organization GLOBOCAN 2012. Estimated cancer incidence, mortality and prevalence worldwide in 2012.

[25] World Health Organization Ebola data and statistics.

[26] World Health Organization Immunization surveillance, assessment and monitoring. Data, statistics and graphics by subject (disease incidence table downloaded)..

[27] Global Burden of Disease Study (2016). Mortality and Causes of Death Collaborators. Global, regional, and national life expectancy, all-cause mortality, and cause-specific mortality for 249 causes of death, 1980?2015: a systematic analysis for the Global Burden of Disease Study 2015. Lancet.

[28] Global Burden of Disease Study (2016). Global, regional, national, and selected subnational levels of stillbirths, neonatal, infant, and under-5 mortality, 1980-2015: a systematic analysis for the Global Burden of Disease Study 2015. Lancet..

[29] Global Burden of Disease Study (2016). Global, regional, and national levels of maternal mortality, 1990-2015: a systematic analysis for the Global Burden of Disease Study 2015. Lancet..

[30] NCD Risk Factor Collaboration (NCD-RisC) (2016). Worldwide trends in diabetes since 1980: a pooled analysis of 751 population-based studies with 4.4 million participants. Lancet..

[31] Streifel C (2015). How did Ebola impact maternal and child health in Liberia and Sierra Leone?.

